# Childhood Vasculitis

**DOI:** 10.3389/fped.2018.00421

**Published:** 2019-01-10

**Authors:** Anja Schnabel, Christian M. Hedrich

**Affiliations:** ^1^Pädiatrische Rheumatologie, Klinik und Poliklinik für Kinder- und Jugendmedizin, Universitätsklinikum Carl Gustav Carus, TU Dresden, Dresden, Germany; ^2^Department of Women's and Children's Health, Institute of Translational Medicine, University of Liverpool, Liverpool, United Kingdom; ^3^Department of Paediatric Rheumatology, Alder Hey Children's NHS Foundation Trust Hospital, Liverpool, United Kingdom

**Keywords:** vasculitis, inflammation, systemic disease, kawasaki disease, granulomatosis with polyangitis, purpura, classification, paediatric

## Abstract

The term vasculitis covers heterogeneous disorders that share the presence of inflammation of blood vessel walls. Immune cell infiltrates can vary significantly and involve granulocytes or mononuclear cells. Vasculitis can be a symptom of other underlying disorders or the underlying cause of organ specific or systemic disease. Classification of childhood vasculitis is based on clinic, the size of predominantly affected vessels, and the histopathology of inflammatory infiltrates. Timely and accurate diagnosis and (where necessary) treatment initiation determine disease progression and outcomes. In light of new developments and the identification of autoinflammatory conditions with vasculitis, new classification tools may be discussed.

## Background

Vasculitis is an umbrella term for various and heterogeneous disorders sharing the presence of inflammation of blood vessel walls. Immune cell infiltrates can vary significantly and involve granulocytes (neutrophils, eosinophils) or mononuclear cells (monocytes/macrophages, lymphocytes). Vasculitis can be a symptom of other underlying disorders (secondary vasculitis: infections, medication intake, malignancies, autoimmune/inflammatory conditions, etc.) or the cause of organ specific or systemic disease. In the latter case, the term “primary vasculitis” is used (1).

**Figure 1:**
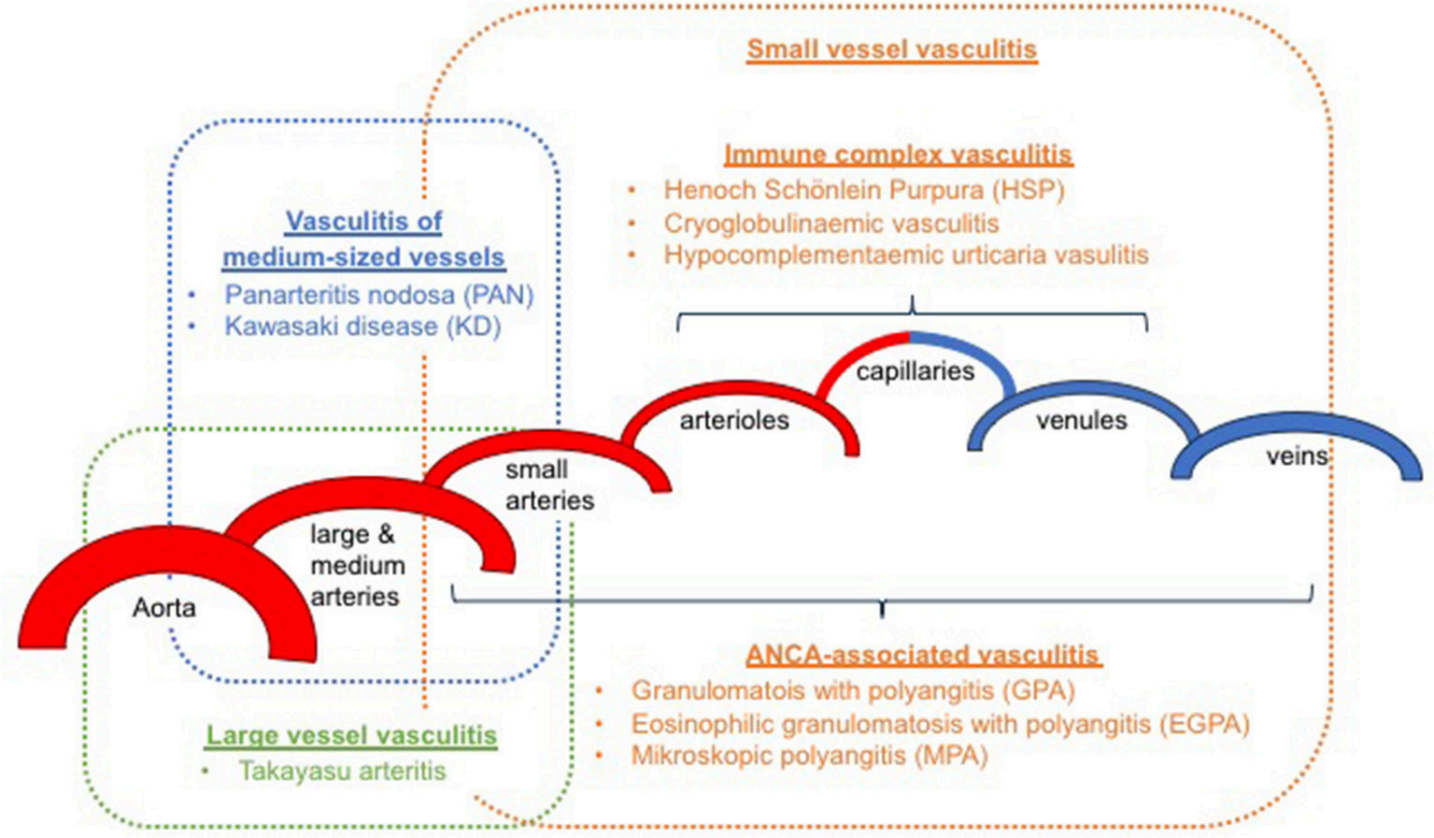
Classification of primary childhood vasculitis based on revised Chapel Hill criteria (2012) and the EULAR/PRES classification (2–5).

It has been an ongoing challenge to classify childhood vasculitis, which is usually based on clinical phenotypes (e.g., single organ vs. systemic vasculitis), the size of predominantly affected vessels (small/medium/large), and the histopathology of inflammatory infiltrates (e.g., granulomatous vs. non-granulomatous). The most commonly applied classifications are the updated Chapel Hill consensus conference criteria for systemic vasculitis (2012) (2), which have not been developed specifically for children, and the EULAR (European League against Rheumatism)/PRES (Pediatric Rheumatology European Society)/PRINTO (Pediatric Rheumatology International Trials Organization) classification criteria for childhood vasculitis (3–5). Both sets of criteria are largely identical for primary systemic vasculitis (Figure 1) (with the exception for giant cell arteritis), but show some differences for the classification of secondary forms (Table 1) and vasculitis limited to single organ systems.

**Table 1:** Selection of underlying causes in secondary vasculitis.

**Cause**	**Example**
Infections	Viral: *Hepatitis A/B/C, HIV, CMV*
	Bacterial: *Neisseria, mycobacteria*
	Fungal: *Candida, aspergillus*
Malignancies	Lymphoma, lymphoproliferative syndrome, etc.
Autoimmune	SLE, MCTD, JDM, IBD, etc.
Medication, drugs	Serum sickness, G-CSF, amphetamines, cocaine, heroin, etc.
Others	Multiple

*HIV, Human immunodeficiency virus; CMV, Cytomegaly virus; SLE, Systemic lupus erythematosus; MCTD, Mixed connective-tissue disease; JDM, Juvenile dermatomyositis; IBD, Inflammatory bowel disease; G-CSF, Granulocyte colony stimulating factor*.

This manuscript provides an overview on current classification of childhood vasculitis, focusing on primary systemic vasculitis. We deliver a brief summary on clinical signs, basic pathomechanisms involved and treatment options. More comprehensive discussion of individual forms of vasculitis, their molecular pathophysiology and treatment available is discussed in focused manuscripts of this special topic.

## Large Vessel Vasculitis

Large vessel vasculitis (LVV) mainly affects large arteries and includes giant cell arteritis (2). In childhood, primary LVV is limited to Takayasu arteritis (TA). Temporal giant cell arteritis, that predominantly involves the temporal artery, carotid and/or vertebral arteries, does not occur in children (1, 3, 5). Takayasu arteritis (TA) is characterized by granulomatous vasculitis of the aorta and/or its main branches, but also coronary and/or pulmonary arteries (6, 7). While TA usually manifests in the third or fourth decade, children (including infants) and adolescents can develop disease. The exact incidence and prevalence of TA in childhood is not known and variation has been reported between geographical regions for adults (UK: 0.8/million vs. Asia 2/million; likely 10–20% of who are children) (8, 9). Girls and young women are more frequently affected when compared to boys (3:1) (10). The molecular pathophysiology of TA remains unclear, and genetic factors, humoral, and cellular autoimmunological factors, as well as infections have been discussed as contributors. Chronic inflammation results in scarring and stenosis of large vessels (6, 7).

Clinical presentation of TA is highly variable, ranging from mild symptoms (e.g., arterial hypertension and/or weak peripheral pulses) to cardiac and/or respiratory failure, and further organ involvement (CNS, gastrointestinal system, etc.). Disease stages prior to the development of key clinical symptoms, such as “pulselessness,” are characterized by non-specific symptoms associated with systemic inflammation and include fever, night sweats, malaise, arthralgia, and myalgia. As inflammation progresses, vascular stenosis develop and may result in intermittent claudication, vascular bruits and/or hypertension (11).

**Figure 2:**
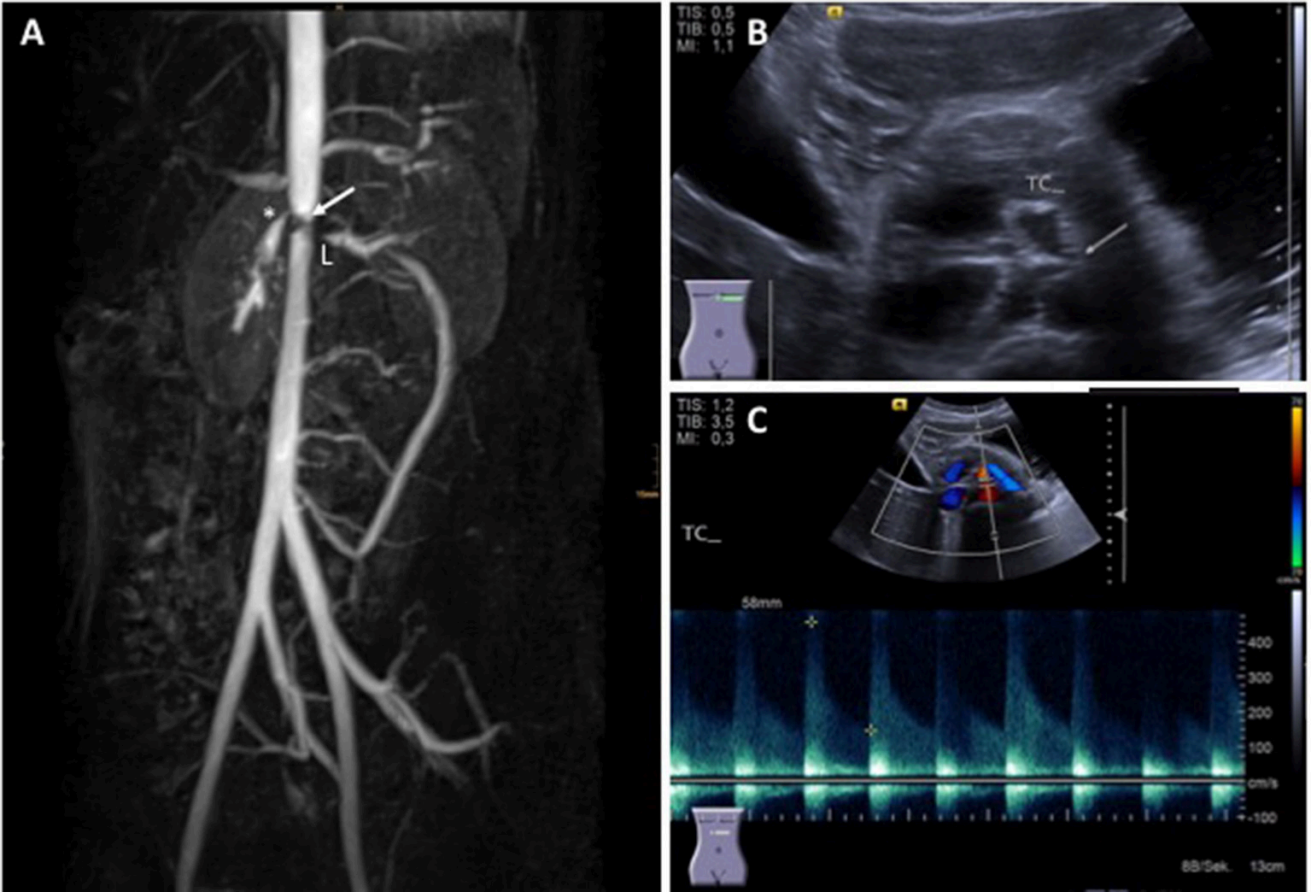
Takayasu arteritis in a 12-year-old girl. **(A)** MRI angiography showing significant stenosis of the coeliac truncus (*) and lower aorta (→); artifacts are caused by stent implants in renal arteries (L) (provided by Dr. Gabriele Hahn, Pädiatrische Radiologie, Universitätsklinikum Carl Gustav Carus, TU Dresden), **(B)** transversal abdominal ultrasonography indicating significant stenosis of the coeliac truncus, **(C)** Stenosis of coeliac truncus resulting in pathologically increased arterial blood flow velocity (provided by Dr. Heike Taut, Klinik und Poliklinik für Kinder- und Jugendmedizin, Universitätsklinikum Carl Gustav Carus, TU Dresden). Consent of the patient was obtained to publish these images.

Diagnosis is usually based on EULAR/PRINTO/PRES classification criteria (Table 2) and informed by imaging (usually MRI angiography; Figure 2). Biopsies may be helpful in unclear cases (10, 12, 13).

**Table 2:** EULAR/PRINTO/PRES criteria for TA.

**Pathology in angiography plus one of the following**	**Aorta or main branches, pulmonary arteries: focal-segmental occluding or dilating changes exclude: arteriosclerosis and fibromuscular dysplasia**
(1) Absence of peripheral pulses, claudication	Weak pulses over A. radialis and/or A. ulnaris; muscle pain and/or weakness after minor exercise
(2) Asymmetrical BP	>10 mmHg (systolic) difference between arms
(3) Murmur (70%)	A. subclavia, A. carotis, abdominal aorta
(4) Hypertension	>95. Percentile
(5) Systemic inflammation	ESR > 20 mm/h, elevated CrP

*BP, Blood pressure; CrP, C-reactive protein*.

Treatment is guided by organ involvement and pre-existing damage and is currently not standardized (14). Pharmaceutical treatment involves corticosteroids as first-line induction treatment (15), disease-modifying antirheumatic drugs methotrexate (MTX), azathioprine (AZA), cyclosporine A (CsA), and mycophenolate mofetil (MMF), TNF inhibitors (infliximab/IFX, adalimumab/ADA, etanercept/ETA), IL-6 inhibitors (Tocilizumab), and B cell depleting agents (Rituximab/RTX) (14, 16–19). Furthermore, Cyclophosphamide (CPM) can be discussed but should be reserved for otherwise treatment refractory or acutely life-threatening cases. If hemodynamically relevant vessel stenosis occur, interventional or surgical interventions may be necessary, but should be avoided if possible, since they are discussed to promote inflammation (14).

## Medium-Sized Vessel Vasculitis

This group includes two forms of primary childhood vasculitis: Kawasaki disease and polyarteritis nodosa.

Kawasaki disease (KD) is a systemic necrotizing vasculitis of small and medium-sized vessels (20). A relatively common and severe complication is the involvement of coronary arteries that can result in aneurysms and cardiac infarction. KD is a rare condition in adults. More than 90% of KD cases affect children and infants (21). Globally, KD is the most common primary childhood vasculitis (Japan 239/100,000 children under 5 years), in predominantly Caucasian populations (9/100,000 children under 5 years), Henoch Schönlein purpura (HSP) is more common (see below). The molecular pathophysiology of KD is unclear (22–24). Genetic predisposition (*BLK, CASP3, CD40, FCGR2A*, HLA class II, *IPTKC*), infectious triggers, super antigens, humoral factors and immune complexes have been suggested to contribute. In the acute phase of KD, monocytes/macrophages and T cells produce pro-inflammatory mediators that result in endothelial inflammation and the clinical picture of KD (22, 23).

**Figure 3:**
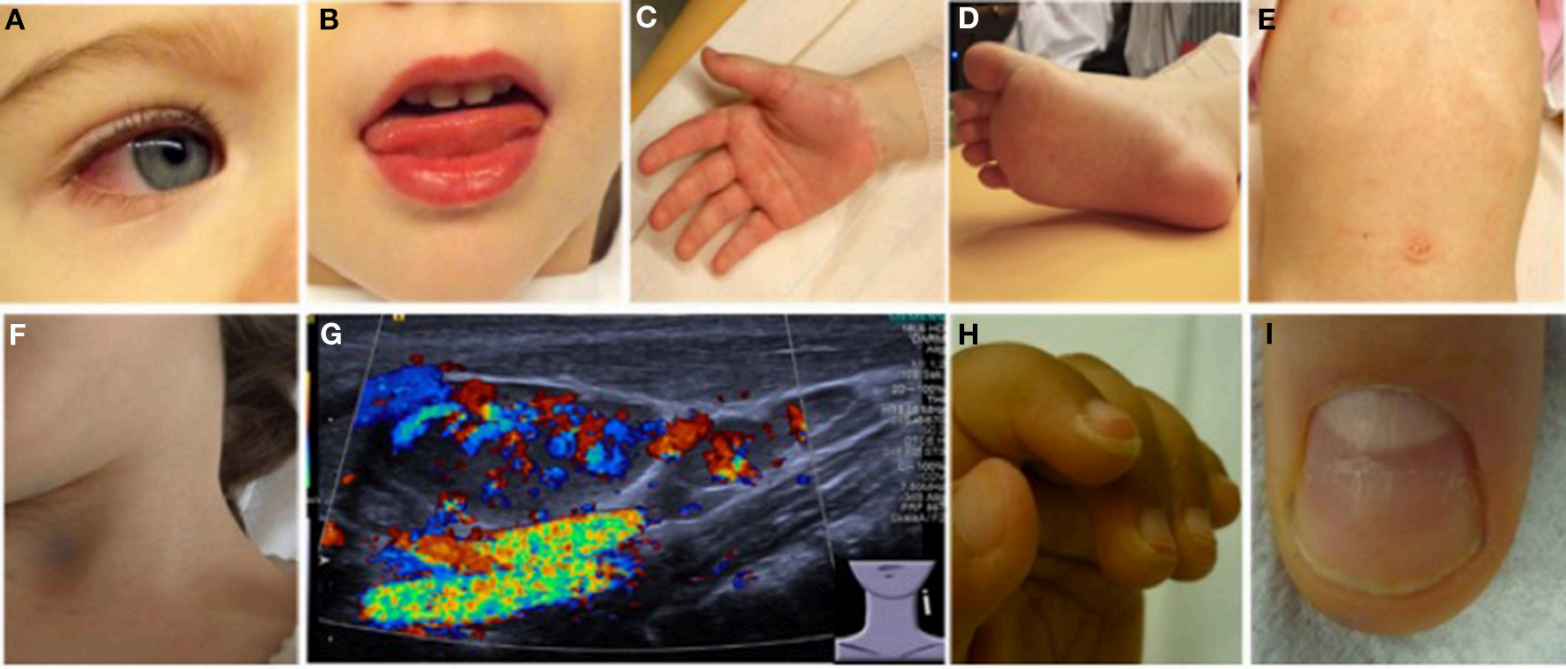
Clinical criteria in KD. **(A)** Bilateral non-purulent conjunctivitis (80–90%), **(B)** changes to oropharyngeal mucous membranes, including injected and/or fissured lips, strawberry tongue (80–90%), **(C)** Palmar, and/or **(D)** plantar erythema **(E)** polymorphous exanthema, primarily truncal, not vesicular (>90%), and **(F,G)** cervical lymphadenopathy (>1.5 cm) (50%). **(G)** Ultrasound of enlarged cervical lymph nodes with increased perfusion (provided by Dr. Heike Taut, Klinik und Poliklinik für Kinder- und Jugendmedizin, Universitätsklinikum Carl Gustav Carus, TU Dresden). **(H)** Periungual desquamation (in covalescent phase) (80%), **(I)** Beau lines; images from (22).

Most (75%) of patients develop KD before their 5th birthday. By definition, fever without focus is present in all KD patients. In children under 5 years-of-age, KD should be considered in the case of unexplained fevers over 4–5 days (22, 23, 25). Infants are less frequently but more severely (increased risk for aneurysm development) affected and tend to exhibit “incomplete” clinical pictures that can be challenging to diagnose (26). The term “atypical KD” is reserved for children who fulfill diagnostic criteria for KD, but experience a not typical disease course, which may, amongst other symptoms include exudative pharyngitis and/or conjunctivitis, aseptic meningitis, arthritis, and/or anterior uveitis. Without timely establishment of treatment, 15–25% of KD patients will develop coronary aneurysms, which define prognosis and long-term outcomes (27). Diagnosis is based on criteria summarized in Table 3 and Figure 3 (28, 29).

**Table 3:** Clinical criteria for the diagnosis of “classical” KD (22).

**Fever of unknown origin for ≥5 days plus 4 of the following if not explained by another condition**. **The diagnosis can also be made on day 4 in the presence of ≥4 principal clinical criteria (particularly when redness and swelling of the hands and feet are present)**
• Bilateral Conjunctivitis (80–90%) •Changes to oropharyngeal mucous membranes, including injected and/or fissured lips, strawberry tongue and enanthema (80–90%) •Palmar and/or plantar erythema and/or periungual desquamation (in convalescent phase) (80%) •Polymorphous exanthema, primarily truncal, not vesicular (>90%) •(Mostly) cervical lymphadenopathy (at least one lymph node >1.5 cm) (50%)

Laboratory findings reflect systemic inflammation and include elevated CRP (≥30 mg/l) and ESR (>40/h), elevated liver enzymes (ALT ≥50 U/l), hypoproteinemia, elevated cholestasis parameters, thrombocytosis, leukocytosis, and/or anemia (22).

Early and aggressive treatment of KD aims at symptom alleviation and the prevention of vasculitis-associated damage (such as aneurysms). Standard treatment includes salicylic acid (initially during febrile period 30–50(−80) mg/kg/day, followed by 3–5 mg/kg/day for 6–8 weeks), and intravenous immunoglobulins (IVIG; usually 2 g/kg over 8–12 h). IVIG should be applied within the first 7–10 days of fever to reduce the risk for coronary aneurysms. Concomitant treatment with prednisolone should be considered in KD patients with a high-risk for the development of coronary aneurysms or individuals who do not respond to a first course of IVIG. In otherwise treatment refractory cases, cytokine-blocking strategies and other have been discussed (22, 23, 27, 28, 30). Evidence for the usefulness of risk scores exists in Asian populations, but their value in predominantly Caucasian populations remains unclear (22, 31–33).

Panarteritis nodosa (PAN) is a necrotizing systemic vasculitis of medium-sized vessels (3). Globally, after KD and HSP, PAN is the third most common childhood primary vasculitis (34). Recently, a rare disorder caused by loss-of-function mutations in the *ADA2* (or formerly *CECR1*) gene encoding for adenosine deaminase 2 (ADA2) has been described and accounts for a subset of particularly early-onset cases of PAN (Deficiency of ADA2: DADA2) (35–37). The molecular pathophysiology of remaining PAN cases is currently unclear. Interestingly, increased incidence and prevalence was recorded in individuals who experience Familial Mediterranean Fever (FMF), one of the most common autoinflammatory disorders in childhood (38). The result of transmural vessel inflammation is scarring and (name giving) nodular changes to vessel walls and aneurysm formation (24). For the diagnosis of PAN, classical angiography and (at least in some patients) angio-MRI can be applied to demonstrate vasal microaneurysms in affected organs (3, 39).

Though generally any organ system can be involved, most patients develop vasculitis of the skin, musculoskeletal system, kidneys and/or gastrointestinal system. Less frequently, the heart and peripheral and/or central nervous system can be affected. Initially, non-specific general symptoms, including fever and malaise are common (5, 34). Diagnosis can be made bases on EULAR/PRINTO/PRES criteria (Table 4) (3).

**Table 4:** EULAR/PRINTO/PRES criteria for PAN (3).

**Criterion**		**Frequency (%)**
**MAJOR CRITERIA (ONE HAS TO BE PRESENT)**		
Histological evidence	Necrotizing vasculitis of small or medium-sized vessels	–
Pathology on angiographic studies	Aneurysm, vessel occlusion (exclude fibromuscular dysplasia or arteriosclerosis)	–
**MINOR CRITERIA (ONE HAS TO BE PRESENT)**		
Skin involvement	Livedo reticularis, painful subcutaneous nodules, skin infarction, digital gangrene	70–90
Myalgia	Myalgia and/or myogelosis	40–70
Arterial hypertension	>95. Percentile	20–35
Peripheral neuropathy	Mononeuritis multiplex, polyneuropathy	20
Kidneys	Proteinuria, hematuria, kidney failure	20

Because of the rarity of childhood PAN, widely accepted and evidence-based treatment recommendations do not exist. Treatment is usually informed by adult rheumatology and includes corticosteroids, IVIG, DMARDS for maintenance treatment, and/or CPM for severe cases (40, 41). Recently, anti-TNF agents have been demonstrated effective at least in a subset of DADA2 patients (35).

## Small Vessel Vasculitis

Small vessel vasculitis (SVV) predominantly affects parenchymal arteries, arterioles, capillaries, and venules. Medium-sized arteries and veins may also be affected. This group can be subdivided into immune complex mediated and ANCA-associated vasculitis.

While Henoch Schönlein purpura (HSP) is the most common form of primary childhood vasculitis in Europe and North America, it is significantly less common in adults (3–14 cases per million) (42). Exact numbers on prevalence and incidence do not exist secondary to very variable presentations and resulting variability in medical needs and providers involved.

**Figure 4:**
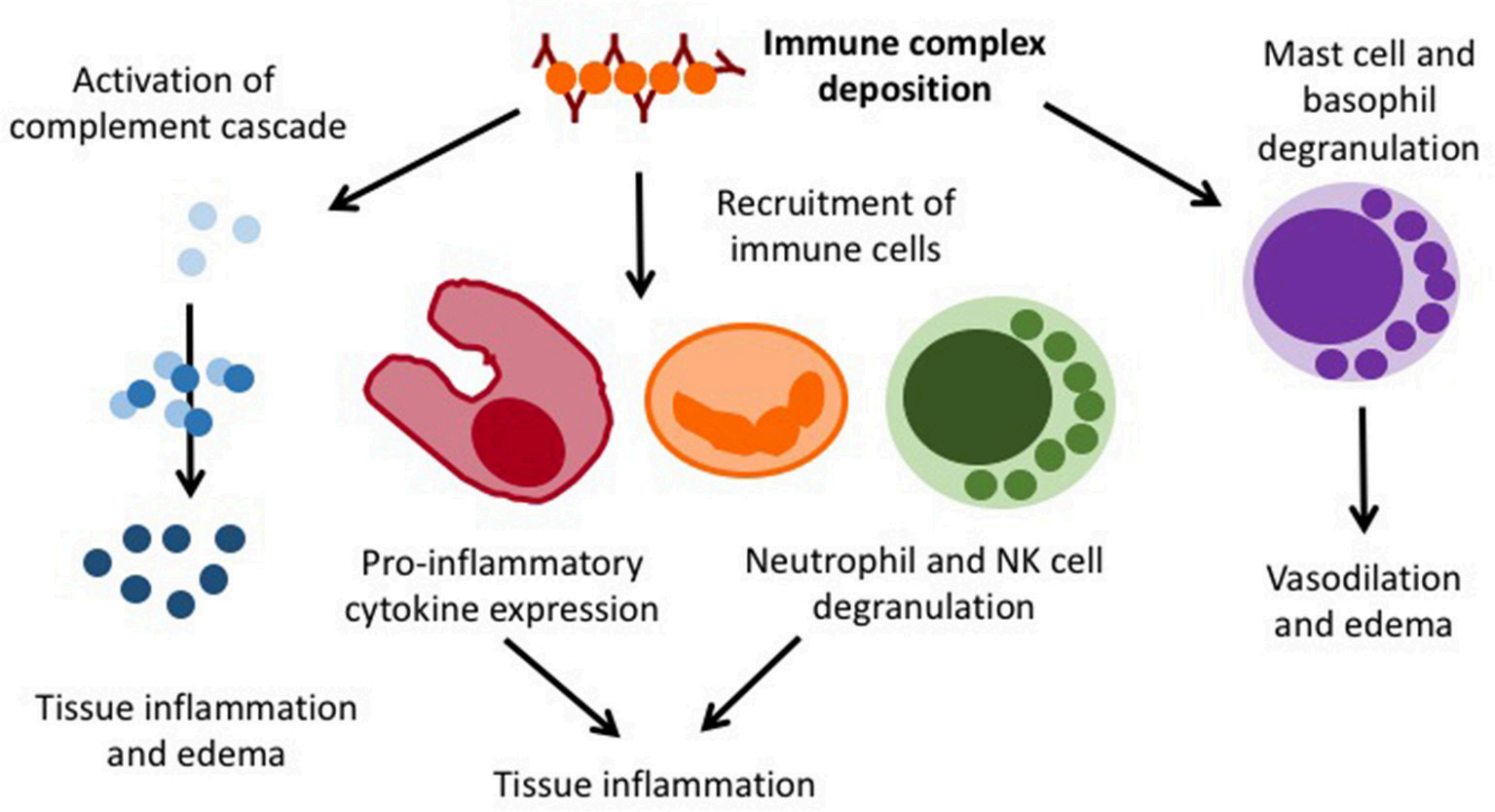
Pro-inflammatory mechanisms in immune complex vasculitis.

Henoch Schönlein purpura (HSP) is an immune complex vasculitis with (mainly) IgA containing immune depositions in small vessels. It can generally affect all age groups (20/100,000/year), but is most common in 4–6 year-old children (70/100,000/year). Boys are slightly more frequently affected as compared to girls (1.2–2:1) (43). While the exact pathophysiology of HSP is unknown, an association with previous infections within the past 2–4 weeks has been recorded (44). As the result of an unknown trigger mechanism, IgA containing immune complexes deposit in small vessels and cause complement activation, immune cell invasion, endothelial activation, and lastly vasculitis (Figure 4).

Henoch Schönlein purpura (HSP) is a diagnosis of exclusion and no specific diagnostic tests are available. Secondary immune complex vasculitis and thrombocytopenic purpura require to be excluded. To diagnose HSP, EULAR/PRINO/PRES classification criteria are commonly used (Table 5) (3). Clinical presentation is variable and, in addition to palpable purpura, includes relatively common symptoms of abdominal pain, arthritis or nephritis, as well as additional organ involvement and associated complications (Table 6). Laboratory findings are unspecific and include (usually) mildly elevated inflammatory parameters (ESR and CrP), normal or slightly reduced serum C3 and/or C4, the absence of high-titer autoantibodies (particularly ANCA and ANA) and, in 50% of all cases, elevated serum IgA and/or IgM (46). Fecal occult blood tests and urinary specimens are recommended at diagnosis. Since glomerulonephritis can manifest after the initial phase, regular urinary analysis at least for the first 6 months after disease-onset are recommended.

**Table 5:** EULAR/PRINTO/PRES criteria for HSP (3).

**Criterion**		**Frequency (%)**
Palpable purpura plus one of the following	- Symmetric purpura, mainly over extremities following gravity - Exclude thrombocytopenia!	100
Abdominal pain	- Colic-like, postprandial - Nausea, gastrointestinal bleeding - Intussusception, infarction, perforation	50
Histopathology	- Immune complex vasculitis - Proliferative glomerulonephritis (IgA deposition)	–
Kidney involvement	- Proteinuria >0.3 g/24 h or - Albumin/creatinine ratio >30 mmol/mg - Micro-hematuria - Arterial hypertension - Nephritic or nephrotic syndrome	20–40
Joint involvement	- Arthritis, mostly ankles and/or knees	70

**Table 6:** Non-cutaneous complications in HSP (45).

**Complication**		**Frequency (%)**
Urogenital	- Testicular bleeding - Orchitis, urethral stenosis	5–10
CNS	- Coma, ataxia, intracerebral bleeding - DD: acute hypertensive crisis as a result of kidney failure	2
Lungs	- Reduced gas exchange capacity, pulmonary bleeding	<1

Treatment depends on clinical presentation and organ involvement. Non-steroidal anti-inflammatory drugs (NSAIDs) or acetaminophen/paracetamol can be considered for analgesia in cases with arthritis or arthralgia. For gastrointestinal involvement treatment with corticosteroids (usually 1–2 mg/kg/day for 1 week, followed by taper over 2–3 weeks) can be considered. However, intestinal perforations have been seen under treatment (47, 48). Substitution of factor XIII can be discussed for gastrointestinal bleedings. Of note, studies indicate that early corticosteroids are not effective in preventing HSP-associated nephritis (45, 49). Evidence for treatment is weak, and expert recommendations have been provided e.g., by the German Society for Pediatric Nephrology (GPN) (Table 7) (50). Bed rest can be considered in severe cases.

**Table 7:** Treatment recommendations for severe HSP nephritis (50).

	**1 (mild)**	**2 (moderate)**	**3 (nephrotic syndrome)**	**4 (kidney failure)**
**HSP STADIUM AND TREATMENT OPTIONS**				
Proteinuria (g/g creatinine)	<2	>2		
GFR (ml/min/1.73 m^2^)	>90			< 90
Serum albumine (g/dl)	>2.5		< 2.5	
Kidney histology	–	No membrane proliferation	Active nephritis, membrane proliferation	
Therapeutic options	–	Ramiprile	Ramiprile + Methylprednisolone potentially additional immune suppression	Ramiprile + Methylprednisolone + Cyclophosphamide potentially additional immune suppression

The group of ANCA-associated vasculitis includes granulomatosis with polyangitis (GPA, formerly Wegener's granulomatosis), eosinophilic granulomatosis with polyangitis (EGPA, formerly Churg-Strauss syndrome), and microscopic polyangitis (MPA). All of which are characterized by destructive vasculitis of small and medium-sized arterial vessels, the presence of ANCA antibodies, and multi-organ involvement (1, 3, 4).

**Figure 5:**
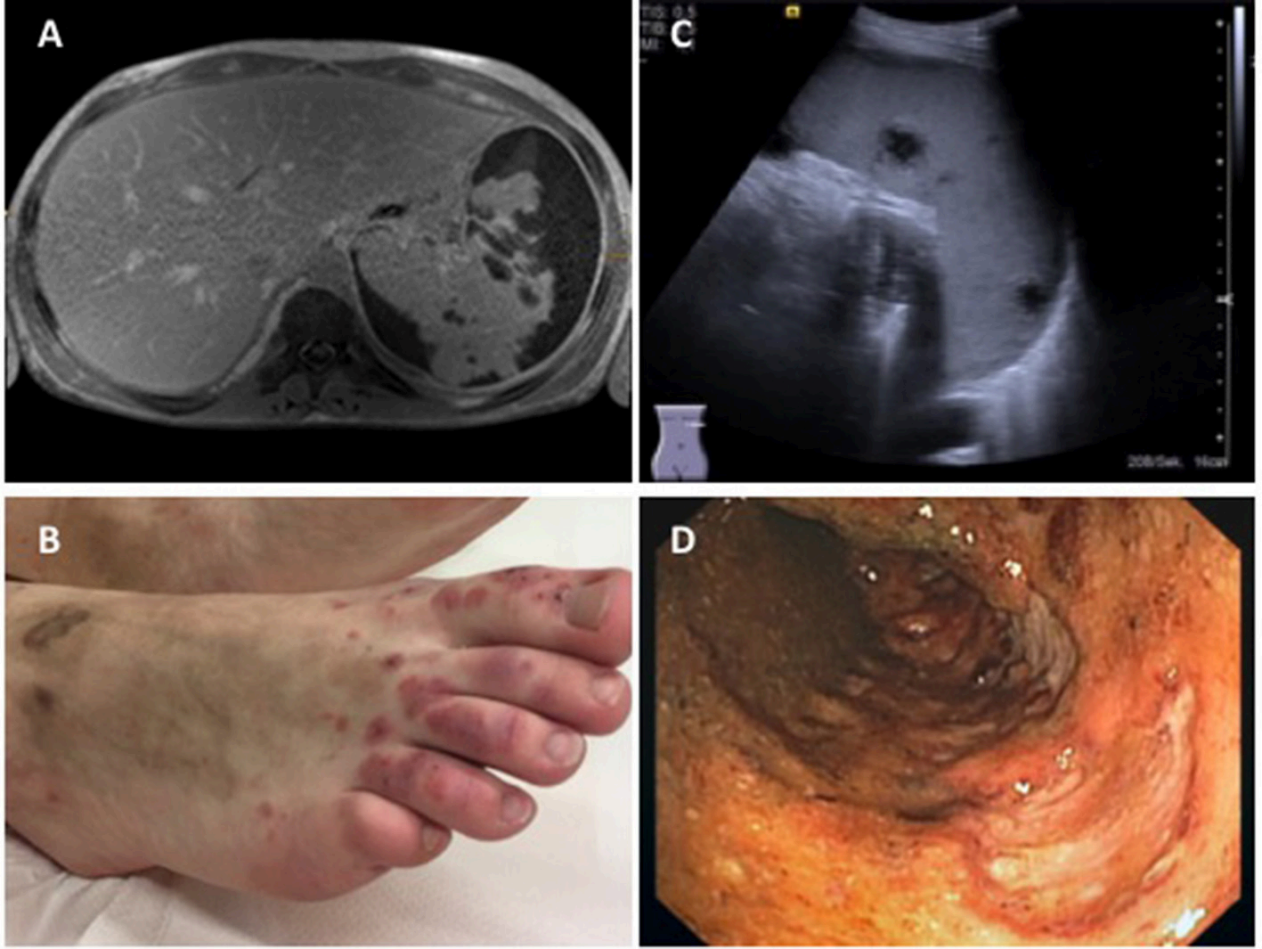
Findings in a 16-year-old boy with GPA. **(A)** Abdominal MRI showing pronounced parenchymatous necrosis of the spleen with distension of the spleen capsule (provided by Dr. Gabriele Hahn, Pädiatrische Radiologie, Universitätsklinikum Carl Gustav Carus, TU Dresden); **(B)** Necrotizing skin vasculitis on legs and feet; **(C)** Splenomegaly, spleen with increased echogenicity, subcapsular fluid accumulation, hypoechogenic necrotic areas with absence of perfusion (provided by Dr. Heike Taut, Klinik und Poliklinik für Kinder- und Jugendmedizin, Universitätsklinikum Carl Gustav Carus, TU Dresden); **(D)** Severe enterocolitis (shown: transversal colon) with deep ulcerations as a result of necrotizing vasculitis (provided by Dr. Martin Laass, Klinik und Poliklinik für Kinder- und Jugendmedizin, Universitätsklinikum Carl Gustav Carus, TU Dresden). Consent of the patient was obtained to publish these images.

Granulomatosis with polyangitis (GPA) is rare in children. Its incidence is estimated to be around 1/1,000,000. The mean age at disease-onset is around 14 years. General symptoms, including fevers, weight loss, and fatigue are present in over 90% of all patients. Diagnosis is usually made based on EULAR/PRINTO/PRES classification criteria (Table 8) (3), and additional symptoms can include arthritis, erythema, ulcerations, and gastrointestinal involvement (Figure 5) (51).

**Table 8:** EULAR/PRINTO/PRES criteria for GPA (3).

**Criterion**		**Frequency (%)**
1. Histopathology	- Granulomatous vasculitis of small vessels	–
2. ENT	- Chronic rhinitis/sinusitis - Oral and/or nasal ulceration - Nasal septum defect, saddle nose - Mastoiditis, hearing loss, recurrent epistaxis	80
3. Laryngo-tracheo- bronchial system	- Subglottic, tracheal, bronchial stenosis	40
4. Lungs	- Cough, dyspnea, obstruction - Pulmonary bleeding - Granuloma, cavernosis, infiltrates in X ray or thorax CT	80
5. Kidneys	- Proteinuria > 0.3 g/24 h or - Albumin/creatinine ratio >30 mmol/mg - Hematuria - Necrotizing glomerulonephritis	75
6. ANCA antibodies	- ANCA positivity (immune florescence, ELISA)	90

Untreated, GPA has a mortality close to 100% within the first year, and regardless of treatment, up to 60% of patients experience subsequent disease flares. When compared to GPA in adults, children, and young people more frequently experience multi-organ involvement, kidney involvement, subglottic stenosis and nose deformities (51, 52).

Eosinophilic granulomatosis with polyangitis (EGPA) is a necrotizing vasculitis characterized by eosinophilic infiltrates in small and medium-sized vessels (53, 54). Reliable demographic information and/or classification criteria for the pediatric age-group do not exist, and diagnosis is informed by ACR criteria for adults with GPA (53). For classification as EGPA, four of six criteria must be fulfilled: (i) Asthma, (ii) Eosinophilia (peripheral blood) >10%, (iii) Mono- or polyneuropathy, (iv) pulmonary infiltrates, (v) para-nasal sinus anomalies, and (vi) biopsy with extra vascular eosinophilic infiltrates.

The exact pathophysiology of EGPA remains unclear. Inappropriate Th2 activation and IL-4, IL-5, and Il-13 expression play a central role and reflect parallels to allergic disease. Progressive immune dysregulation triggers a prodromal state that can last several years and include chronic sinusitis, allergies, and severe corticosteroid-dependent asthma. Later, sinusitis, pulmonary infiltrations and sometimes severe gastrointestinal, cardiac, skin manifestations (Livedo, painful subcutaneous lesions, infarctions, etc.), and peripheral neuropathy can be present (54, 55).

Microscopic polyangitis (MPA), in contrast to GPA and EGPA, is a non-granulomatous necrotizing vasculitis of small vessels (56). Immune complex deposition is limited or absent (pauci-immune vasculitis). Most patients develop pulmonary (but not upper respiratory tract) involvement with pulmonic hemorrhage and sometimes kidney involvement (27%). Additional organ systems, including the gastrointestinal system, CNS, skin, musculoskeletal system, and end eyes can be affected. Diagnostic or classification criteria for childhood MPA do not exist. Thus, Microscopic polyangitis (MPA) remains a diagnosis of exclusions and is informed by adult rheumatology. The presence of ANCA can be helpful (in adult cohorts: >50% pANCA, 40% cANCA positive) (56, 57).

Treatment of childhood ANCA-associated vasculitis is guided by disease severity. Generally, patients should receive more or less aggressive induction treatment to control inflammation (high-dose prednisolone or i.v. methylprednisolone pulses ± CPM) that is followed by maintenance treatment with more tolerable therapeutic agents (MTX, AZA, MMF) (58). Based on studies in adult cohorts, RTX can be discussed as alternative induction treatment in some cases and appears to be superior to CPM in GPA flares after initial induction treatment with CPM (59, 60). Trimetoprim/sufamethoxazole should be considered for *pneumocystis* prophylaxis. Furthermore, flare reduction has been described in patients with GPA and *S. aureus* colonization (61, 62). In devastating and treatment-refractory cases, autologous stem cell transplantation can be considered (63).

## Vasculitis Affecting Variable Vessel Sizes

Several forms of vasculitis can affect vessels of variable types and diameters.

Behcet's disease (BD) is characterized by inflammatory lesions in vessel walls of all sizes, which may lead to endothelial damage, thrombosis, and aneurysms (64). Chronic recurrent oral and/or genital ulcers occur can be accompanied by additional cutaneous (erythema nodosum, cutaneous pustular vasculitis, etc.), ocular (posterior uveitis, retinal vasculitis), articular (non-erosive poly- or oligo-arthritis), gastrointestinal (abdominal pain, nausea, diarrhea, etc.), and/or central nervous symptoms (aseptic meningitis, vascular thrombosis) (1). Cases of BD can be seen across the globe and in all ethnicities (64). However, prevalence is highest in countries along the Silk Road, where it ranges between 77 and 100/100,000 individuals (0.1–15.9/100,000 in Western Europe) (65, 66). While most patients develop symptoms in young adulthood, 5–10% exhibit childhood-onset BD (67). The pathophysiology of BD is incompletely understood, but genetic associations are likely involved and may be influenced by environmental factors (13, 68, 69). HLA-B51/B5 allele carriers have considerably high risk for BD indicating a possible gene-dose effect (70). Diagnosis can be challenging, especially since children and young people frequently do not develop the full clinical picture of BD and progress over time (64, 71). More than 15 sets of classification or diagnostic criteria have been published (72). Based on clinical differences between age-groups, recently, pediatric classification criteria have been suggested (1, 73). Treatment of BD can be complex and should be informed by clinical symptoms and disease severity. Topical treatment (steroids and/or sucralfate) and systemic treatments (corticosteroids, colchicine, AZA, CsA, thalidomide, apremilast, TNF inhibitors, etc.) are discussed elsewhere (1, 64).

Cogan syndrome (CS) is characterized by predominantly large vessel vasculitis, but can affect any vessel size (1). CS is an extraordinarily rare multisystem inflammatory condition that can involve eyes (keratitis, uveitis, episcleritis) and inner ears (sensorineural deafness, vestibular dysfunction) (2, 74). Unspecific systemic symptoms occur in 50% of all patients, including arthralgia and manifestations of medium-size and small vessel vasculitis. To date, only few pediatric patients have been reported (75). Based on the rarity and lack of pathophysiological understanding of the disorder, data on effective treatments are lacking. Available reports favor DMARDs (AZA, MTX) in combination with TNF inhibitors (75).

## Single Organ Vasculitis

Primary organ vasculitis covers a range of particularly rare disorders characterized by vasculitis of a single organ in the absence of signs indicative of systemic vasculitis (1). Various organ systems can be involved, including the CNS (primary large or small vessel CNS vasculitis) (76, 77), primary testicular vasculitis (78), cutaneous leukocytoclastic vasculitis (1), etc.

## Vasculitis in the Context of Autoinflammatory Disease

Autoinflammatory disorders are characterized by systemic or organ-specific inflammation that is (at least initially) caused by dysregulation of the innate immune system (79, 80). Vasculitis can be a feature seen with several autoinflammatory conditions. Indeed, in some autoinflammatory disorders, including aforementioned BD (1, 81), previously discussed DADA2 (35–37), primary type I interferonopathies STING-associated vasculopathy with onset in infancy (SAVI) (82) and Aicardi Goutières syndrome (83), and haploinsufficiency of H20 (HA20) (84), vasculitis can be the dominant feature. Autoinflammatory conditions are still relatively “new” to the field of Rheumatology and underlying pathomechanisms of systemic inflammation and/or vasculitis remain unclear in many cases. Thus, (with the exception of BD) vasculitis in the context of autoinflammatory disease is not part of currently available classifications for vasculitis, which will likely change in the years to come.

## Conclusions

Vasculitis are rare conditions in children and young people that can be subdivided and classified based on clinical phenotypes (e.g., organ-specific vs. systemic) underlying causes (primary vs. secondary disease), histological patterns (granulomatous, non-granulomatous, necrotizing, etc.), and primarily affected vessel sizes (Chapel Hill and EULAR/PRES classifications: small/medium/large). Timely and accurate diagnosis and (where necessary) treatment initiation are essential, provided the variable severity and outcomes of individual forms of vasculitis. In light of new developments [including the identification of genetic causes, sometimes resulting in expansion of disease-associated phenotypes (e.g., DADA2)] and the identification of autoinflammatory conditions with vasculitis as key feature [including complex genetic BD, but also monogenic disease (DADA2, SAVI, HA20)], new classification tools may be justified in the near future.

## Author Contributions

AS and CH equally contributed to all stages of this manuscript, including conception and writing of the manuscript. AS and CH contributed to manuscript revision, read, and approved the submitted version.

### Conflict of Interest Statement

The authors declare that the research was conducted in the absence of any commercial or financial relationships that could be construed as a potential conflict of interest.
